# Supplementary material for: Growth patterns of children under 5 years old in rural area northern of Abha, Aseer Region, Saudi Arabia
Source: PLoS One. 2024 Feb 23;19(2):e0297279. doi: 10.1371/journal.pone.0297279 (PMC10889841; doi:10.1371/journal.pone.0297279)
Supplement: S1 File — (DOCX) [file pone.0297279.s001.docx]

**Child Growth data sheet**

PHC name (……………..…)

1-Child name (optional )

2-MHN(optional ):

3 Age : y: m :

4- Gender : M F

5- WT currently : KG G

6-Height : cm

7- ORDER OF CHLID : ( NUMSERED )

Number of siblinig in family :

How many male: ………… how many female :……….

8 - Type of feeding :

1. Breast exclusive No Yes if yes , how many month ( )
2. Bottled excluseved No Yes If yes , how many months ( )
3. Mixed No Yes If yes , how month ( )

Thanks

Filled person :

Name : Specialty :

NB : Parents or guardians consent was signed No Yes ,if yes attached consent

**موافقة على اجراء بحث**

**(Consent form in English & Arabic language )**

**I don't against taking information about my son/daughter/his dependent (his data below).**

**Name:**

**Date of birth:**

**which relate to length, weight, date of birth and type of nutrition either breastfeeding or otherwise and duration only for research closure and without any other procedures and be used only for research purposes while maintaining full confidentiality.**

**Research Name: Children's Growth Study for Five Years in Rural Areas in Abha Region**

**Search Supervisor: Safar Abadi Alsaleem**

**Email:asalslim@kku.edu.sa**

**اقر انا السيد /السيدة .......................................................................................................................**

**بانه لامانع من اخذت معلومات عن ابني /ابنتي / من اعيله (بياناته ادناه)**

**الاسم :**

**تاريخ الميلاد :**

**والتي تتعلق بالطول والوزن وتاريخ الميلاد ونوع التغذية اما رضاعة طبيعية او غيرها ومدتها فقط للاغلاض البحثية فقط ودون اي اجراءات اخري وان لا تستخدم الا للاغراض البحثية فقط مع الحفاظ على كامل السرية**

**اسم البحث : دراسة النمو لدي الاطفال لاعمار الخمسة سنوات فاقل في الاماكن الريفية بمدينة ابها بمنطقة عسيروعلاقته بطرق التغذية**

**المشرف على البحث : د سفر عبادي ال سليم**

**البريد الالكتروني :**
